# Carbon-Filled Organic Phase-Change Materials for Thermal Energy Storage: A Review

**DOI:** 10.3390/molecules24112055

**Published:** 2019-05-29

**Authors:** Guijun Yang, Yoon-Ji Yim, Ji Won Lee, Young-Jung Heo, Soo-Jin Park

**Affiliations:** Department of Chemistry, Inha University, 100 Inharo, Incheon 22212, Korea; yanggj91@gmail.com (G.Y.); yj_yim@naver.com (Y.-J.Y.); lj529@naver.com (J.W.L.); heoyj1211@gmail.com (Y.-J.H.)

**Keywords:** organic phase change materials, carbon materials, thermal energy storage

## Abstract

Phase-change materials (PCMs) are essential modern materials for storing thermal energy in the form of sensible and latent heat, which play important roles in the efficient use of waste heat and solar energy. In the development of PCM technology, many types of materials have been studied, including inorganic salt and salt hydrates and organic matter such as paraffin and fatty acids. Considerable research has focused on the relationship between the material structure and energy storage properties to understand the heat storage/emission mechanism involved in controlling the energy storage performance of materials. In this study, we review the application of various carbon-filled organic PCMs in the field of heat storage and describe the current state of this research.

## 1. Introduction

In general, new renewable energy such as solar, marine, and wind energy has stochastic volatility and intermittent and thus places high demands on energy storage technology. In recent years, research related to thermal energy storage by sensible heat and latent heat has increased and now plays a major role in practical applications [1]. Among the many thermal energy storage methods, solid–liquid phase-change materials (PCMs) for melting latent heat are an effective method and have received extensive attention because of their high heat storage density and temperature fluctuation, which is far less than the detectable option [2,3,4].

Numerous materials have been tested and implemented as PCMs. These materials can be roughly divided into two types: inorganic and organic PCMs [5]. Paraffin wax, which is a widely used organic PCM, is composed of a group of alkane mixtures characterized by subcooling and favorable thermal and chemical stability, in addition, its actual melting point depends on the chain length, the shorter the chain length, the higher the melting point [6]. Paraffin is advantageous for thermal energy storage (TES) applications, as it can be used at low-to-medium temperatures. However, the lower thermal conductivity of paraffin wax can considerably reduce the energy charge and discharge rates. To improve the thermal conductivity of low-thermal conductivity paraffin (or other PCMs), an effective method is to add highly conductive particles [7]. Composite PCMs that incorporate thermally conductive fillers are continually being researched and developed [8,9]. Such fillers have included metallic beads/powders/particles [10,11,12,13,14,15,16,17,18,19,20,21,22,23,24,25], carbon fibers [26,27], expanded graphite [28,29,30,31,32,33], carbon nanofibers (CNFs) [34,35,36,37], carbon nanotubes [38,39,40,41,42,43,44,45,46], and graphene/graphite nanoplatelets [47,48,49,50,51,52,53].

Of the various thermally conductive fillers that have been investigated, carbon materials are preferred because of their high thermal conductivity and relatively low density [54,55,56,57,58,59,60]. The effects of the addition of various carbon fillers on the thermal conductivity and energy storage properties of organic PCMs have been widely reported, but few studies have directly compared their performance.

The purpose of this study is to present the current status of the field of research related to organic PCMs that incorporate various carbon materials for application of TES. This study outlines several methods for improving the thermal properties of organic PCMs and describes in detail related studies to improve the thermal conductivity of organic PCMs. This study will, thus, help the reader to understand the current technologies, potential problems, and future developmental direction of this research.

## 2. PCMs

### 2.1. Definition of PCMs

Solid–liquid PCMs are materials suitable for cold and hot storage and are potential solutions to improve building thermal control because they can store more energy in a latent form than can the typical sensible energy stored by typical building materials [61,62]. Sensible heat storage is the most common method (ΔQ) [63] and can be described by the following Equation (1):

ΔQ = C ΔT = m c·Δ T(1)

where C is the heat capacity, m is the mass of the material, c is the specific heat of the material, and ΔT is the difference between the final and initial temperatures. Different materials have different heat capacities C, which causes sensible heat to be affected by material properties and application temperatures [63]. Latent heat is another means of storing thermal energy. This method utilizes a material phase-change process to store thermal energy as potential energy. Usually, PCMs undergo a volume change of less than 10% during the phase change process [64].

**Figure 1:**
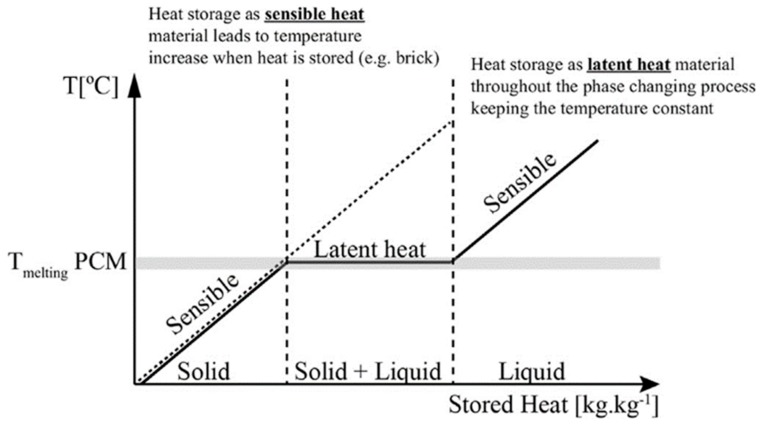
Latent heat storage for the solid–liquid case [65].

Beyond the phase-change process temperature region, PCMs behave like typical materials and store sensible heat. The two major application areas of PCMs can be described as follows (see Figure 1) [65]. First, temperature control: The PCM can absorb and release thermal energy without significant temperature changes, indicating that PCMs can be used to maintain temperature stability. And second, the temperature change during energy storage and supply is small.

As Figure 1 shows, in the case of solid–liquid phase changes, when the temperature rises to the melting temperature of the phase-change material, the PCM remains at a constant temperature and the stored heat increases. The latent heat storage (LHS) of the solid–liquid case maintains a constant temperature during the entire phase-change state of the storage material. When the temperature is higher or lower than the phase change that occurs in the temperature range, the heat is stored in the form of sensible heat, and the temperature of the material rises. During the endothermic process, the PCM material transforms from a solid to a liquid phase and absorbs energy, and during the exothermic process, the liquid PCMs transform to the solid phase again [66]. The heat of storage in the phase-change process can be calculated from the enthalpy difference (ΔH) between the solid and liquid phases, as shows in the following Equation (2):

ΔQ = ΔH(2)

### 2.2. Classification of PCMs

**Figure 2:**
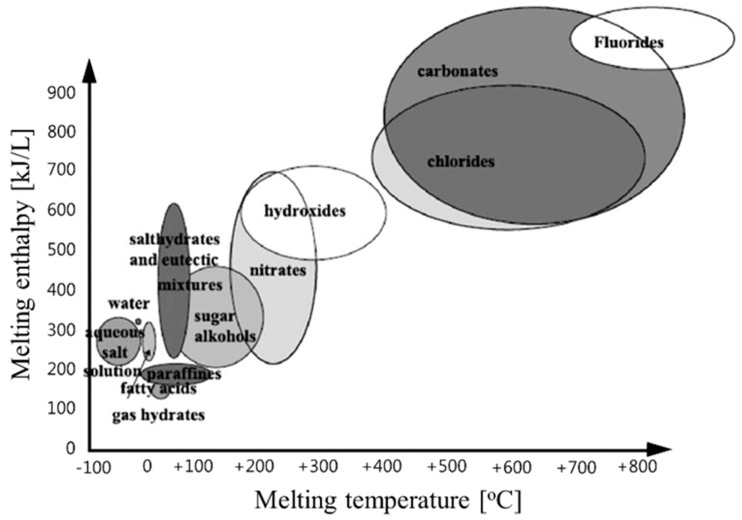
Classes of materials that can be used as thermal storage materials [67].

**Figure 3:**
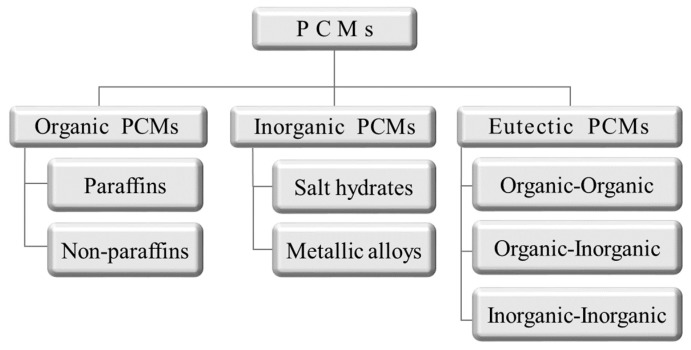
Scheme of phase-change material (PCM) classification [71].

Over the last several decades, many studies have been conducted on energy storage materials, including hydrated salts, paraffins, fatty acids, and eutectic mixtures of organic and inorganic compounds (see Figure 2) [67]. According to their composition, energy storage materials are divided into two categories: organic and inorganic. These two categories can be further divided into two subcategories based on eutectic and mixed temperature ranges (see Figure 3) [68,69,70,71].

Commonly used PCMs include organic PCMs such as paraffin, fatty acids, and polyethylene glycol (PEG) [72,73,74,75]. Commercial paraffins are inexpensive and have considerable storage density (200 kJ/kg or 150 MJ/m^3^). Their supercooling effect is negligible, and they have stable chemical and physical properties as well as good thermal stability. Phase-change materials with these characteristics can be used in applications under a wide range of temperatures, for example, in the construction sector [5]. However, paraffin has a low thermal conductivity, that is, with an average of 0.2 W/mK, which is much lower than some common organic PCM solutions (0.8 W/mK).

## 3. Organic PCMs with Carbon Fillers

### 3.1. Organic PCMs

#### 3.1.1. Paraffin

Paraffin wax has received considerable attention in thermal energy storage because of its good thermal physical properties, including a suitable melting temperature, high latent heat energy, negligible supercooling, and stable chemical and thermal performance [70]. Paraffin wax (C_n_H_2n+2_) generally has a linear, cyclic, or branched structure. The melting point of paraffin wax is between 30 and 90 °C, and its specific melting enthalpies are 180–270 kJ/kg, which is determined by the chain length of the alkane. In general, the melting point of these types of materials increases with increasing average molecular weight, as shown in Table 1 [2,76,77].

#### 3.1.2. Fatty Acids

Fatty acids (CH_3_ (CH_2_)_2n_COOH) are renewable PCMs and are considered potential PCMs for heat storage because of their good thermodynamic and kinetic characteristics such as latent heat of fusion, supercooling, thermal and chemical stability, small volume change, and broad melting point range [78]. Unlike paraffins, individual fatty acids have distinct properties, but their melting points and heats of fusion increase moderately as the number of carbon atoms in the fatty acid molecule increases. The melting point of fatty acids ranges from 5 to 70 °C, whereas their latent heats of fusion range from 45 to 210 kJ/kg. Like other organic PCMs, shape-stabilized PCMs increase the likelihood that practical latent heat storage applications can eliminate the obstacles they are experiencing [79]. The thermal physical properties of some saturated fatty acids are shown in Table 2 [2,76,80].

#### 3.1.3. Others

Studies have shown that when acetate trihydrate is added to a binary mixture of urea–sodium acetate trihydrate (U–SAT), the melting point of the modified U–SAT can be increased from 32 to 44.5 °C without additives [81]. Kaizawa et al. [82] used different sugars and SAT as PCMs, and their thermophysical properties were studied. In addition, the heat transfer performance between the selected PCM and heat transfer oil was also studied. The results show that erythritol has the highest latent heat of 344 kJ/kg at 117 °C and a high decomposition point of 160 °C. It has good chemical stability under repeated phase-change cycles and is the most suitable PCM for practical systems.

Zhao et al. [83] tested the compatibility of SAT with aluminum alloy and copper for 270 days. The results show that the corrosion effect is negligible; aluminum alloy and copper can be used with the SAT for a long time. Sodium acetate trihydrate modified with 2 wt% disodium hydrogen phosphate dodecacarbonate (DHPD) and 2 wt% carboxymethyl cellulose (CMC) as additives has the best performance in reducing supercooling performance. The results show that the thermal charging rate mainly depends on the thermal power level, and the foamed copper/SAT composite PCM with less thermal conductivity enhancer has better heat storage performance. It can be seen from the heat removal process that the subcooling capacity of the thermal storage unit is still greater than the improved SAT.

Canik and Alkan et al. [84,85] prepared a variety of amide functionalized PCMs by condensing carboxylic acid chloride with hexamethylenediamine. With the increase in C atoms in the carboxyl group, the phase transition temperature and enthalpy of hexamethylene dilauroyl, dimyristoyl, and dipalmitamide increased correspondingly, showing a good thermal storage property and prospects for wide application. Kenar et al. [86] proposed a novel organic PCM with oleate bio-based materials through a carbonate exchange reaction between C10-C18 fatty alcohol and dimethyl carbonate or diethyl carbonate under the action of a catalyst. Currently, oleochemical carbonates have yet to study in detail regarding their applicability as PCMs, but the experimental results show that they have obvious phase transition and good latent heat performance. They represent a promising candidate as a renewable PCM that complement the use of fatty acids, fatty alcohols, and fatty acid esters in energy storage.

### 3.2. Thermal Properties of Organic PCMs Filled with Carbon Materials

#### 3.2.1. Thermal Properties of Organic PCMs Filled with Carbon Nanotubes

Carbon nanotubes (CNTs) are a lightweight, high-thermal conductivity nanomaterial with broad heat transfer application prospects, which are commonly used as additives to improve the TES of paraffin and soy wax [87]. Phase-change materials were prepared by melting the waxes and mixing them with different content of CNTs. Experimental results show that CNTs can effectively improve the thermal conductivity of PCMs. In addition, to some extent, the thermal conductivity of PCMs increases with the addition of CNTs. Tang et al. [88] reported a method for producing functionalized multi-walled carbon nanotubes (f-MWCNTs) to enhance thermal conductivity and reduce supercoiling of paraffin PCMs. The carboxylation reaction of n-octadecyl-functionalized f-MWCNTs was conducted by simple H_2_SO_4_ and HNO_3_ mixture, and then salt formation reaction with n-octadecylamine was performed to obtain f-MWCNTs; the MWCNTs improved the thermal conductivity of organic PCMs. It was found that f-MWCNTs can be uniformly dispersed in toluene or paraffin matrix because of the presence of long-chain alkanes in f-MWCNTs. In addition, f-MWCNTs as a nuclear distribution promote heterogeneous nucleation and crystallization of paraffin, which reduces the supercooling of paraffin. Fan et al. [89] reported paraffin-based composite PCMs containing short and long multi-walled carbon nanotubes (S-MWCNTs and L-MWCNTs, respectively) and the thermal properties were also investigated. Of the two types of carbon nanotubes, S-MWCNT showed a relatively higher improvement of 63.49% at a loading of 95 wt%. Xu et al. [90] reported PCMs containing Cu- and Cu_2_O-decorated MWCNTs. Because of the high thermal conductivity of MWCNTs, strong light absorption capacity of Cu_2_O, and the modification of Cu and Cu_2_O on the surface of the nanoparticles, the interfacial thermal resistance between MWCNTs and paraffin is reduced. In their study, the thermal conductivity of each sample was higher than that of pure paraffin. Zhang et al. [91] studied the effect of carbon nanotubes (CNTs) on the thermal behavior of a palmitic stearic acid binary eutectic mixture (PA-SA). Four PA-SA/CNTs composite phase-change material samples with CNT mass fraction of 58 wt% and mass fraction of 1 wt% were prepared. The thermal performance test results show that with the addition of CNTs, the heat release rate of the prepared composite PCMs increases, while the storage rate decreases. The PCMs with higher thermal conductivity, heat release rate, high thermal reliability, and proper thermal properties are a convenient material for thermal energy applications. The thermal properties of some of the prepared organic PCMs with carbon nanotube fillers are listed and compared in Table 3 [87,88,89,90,91,92,93,94].

#### 3.2.2. Thermal Properties of Organic PCMs Filled with Graphite Derivatives

Fan et al. [89] studied the effects of different carbon nanopowders on the thermal conductivity and energy storage performance of paraffin-based nanocomposite phase-change energy storage materials. These include long and short MWCNTs, carbon nanofibers, and graphene nanoplatelets (GNPs). The reported the use of planar graphene nanoflakes (GNPs) to overcome the low-thermal interface resistance of paraffin-based PCMs. Among the four types of carbon fillers, the relative performance of GNPs was increased by 164% at a load of 5 wt% due to the two-dimensional (2D) planar structure, which reduced the thermal interface resistance of the filler/substrate. Xiang et al. [95] investigated the thermal and electrical conductivities of GNP nanocomposites with two sizes and aspect ratios of peeled graphite nanoplates mixed with paraffin wax. They found that when the aspect ratio of the nanoplates was large and their orientation was good, the interface density of the nano-filler was low, and the thermal conductivity and thermal stability of the nanocomposite could be increased. The paraffin wax and exfoliated graphite nanoplates (xGNPs) interacted through van der Waals forces. Discrete singular convolution (DSC) analysis of paraffin nanocomposites with low xGNP concentrations demonstrated that the two peaks corresponding to the melting and crystallization enthalpy of the paraffin wax were not considerably affected by the addition of xGNP particles. In addition, the thermal and chemical inertness of xGNP and the interaction of the xGNP particles with the surrounding matrix enhanced the overall stability of the composite PCM. Comparing the effects of xGNP and graphene on the thermophysical properties of PCM composites, it was found that the graphene-containing composites had lower electroosmotic flow thresholds, and the paraffin conductivity was much higher than that of xGNP-containing composites. However, the thermal conductivity was lower [94].

Wang et al. [96] introduced a novel sebacic acid/expanded graphite (SA/EG) composite phase change material. The optimum mass percentage of SA in the SA/EG composite PCM is about 85%, the composite PCM phase transition temperature is 128 °C, and the latent heat is as high as 187 J/g. The combination of SA and EG effectively prevents the inherent degree of subcooling of the SA to be low. Compared with SA, SA/EG-filled PCM composites show the advantage of negligible supercooling, better thermal reliability and stability. The SA/EG composite PCM can be easily formed into various shapes by dry pressing, and the thermal property loss is small, but the thermal conductivity is remarkably improved. Zhou et al. [97] used an octanoic acid–myristic acid binary eutectic mixture (OA–MA) as a base liquid. A novel composite PCM having an optimum mass ratio (OA–MA:EG = 93:7) was prepared by uniformly adsorbing OA–MA into the porous structure of expanded graphite (EG). The phase transition temperature of OA–MA/EG was 6.8 °C and the latent heat was 136.3 J/g. The addition of EG not only improves the thermal conductivity of OA–MA, but also increases the nucleation rate of OA–MA. After 100 times of cold storage and discharge comparison experiments, the phase transition temperature and latent heat of OA–MA/EG did not change significantly.

Narayanan et al. [78] produced a new type of synthetic PCM by mixing paraffin and oleic acid with a small amount (0.5 wt%) of nano-graphite as a support material using a simple melt-mixing process. Solar water heaters prepared using these types of materials can produce hot water without nighttime or solar radiation, and the prepared gloves can continue to heat up in a low-temperature environment. The material showed inherent properties such as high thermal conductivity, ultra-fast thermal charging, and fast heat transfer characteristics, without considerably reducing the solar energy harvesting, high photothermal conversion, latent heat, or phase transition temperature.

Exfoliated graphite nanoplates were also used as a reinforcing agent in a palatinic acid (PA)/polyaniline (PANI)/xGNP composite to form a stable PCM through ultrasonic treatment. Zeng et al. [98] used xGNPs to enhance the structure, energy storage properties, and thermal conductivity of polyaniline (PANI)-based shape-stabilized PCMs. The content of PA in the prepared stabilized PCMs was fixed at 75% by weight. The results show that xGNPs can significantly improve the thermal conductivity of the prepared shape-stable PCMs under the premise of maintaining heat storage capacity. The PA/PANI/xGnP-type stabilized PCM with a mass fraction of 7.87% has a thermal conductivity of 1.08 W/(m K) and a storage capacity of 157.7 J/g. The thermal conductivity is 237.5% higher than that of the PA/PANI type stable PCM. The prepared PA/PANI/xGnP shape-stable PCM is expected to exhibit better performance in solar thermal applications.

**Figure 4:**
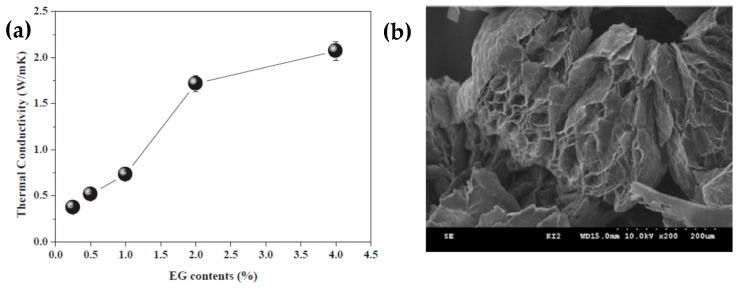
(**a**) Thermal conductivities of the erythritol/expanded graphite composites as a function of the EG content and (**b**) SEM image of the worm-like and porous EG.

Shin et al. [99] prepared an erythritol and exfoliated graphite composite using an impregnation method, which effectively improved the thermal conductivity and thermal cycle stability of erythritol. Composites containing 4 wt% exfoliated graphite exhibited the best thermal performance in the tested SSPCMs, considerably improving the thermal conductivity and thermal cycling stability of 2.06 W/mK (Figure 4a). However, the melting point of the composite material was slightly reduced. After multiple melt-coagulation cycles, the initial latent heat of the composite material was the lowest, but the insect-like morphology and porosity of the EG reduced the heat leakage of the composite material (Figure 4b). Expanded graphite (EG) with different interlayer spacing was combined with molten erythritol using a simple mixed impregnation method to form erythritol/EG PCMs. The effect of the additives on the thermal behavior was investigated for various interlayer distances of EG using the thermal equilibrium technique [100].

**Figure 5:**
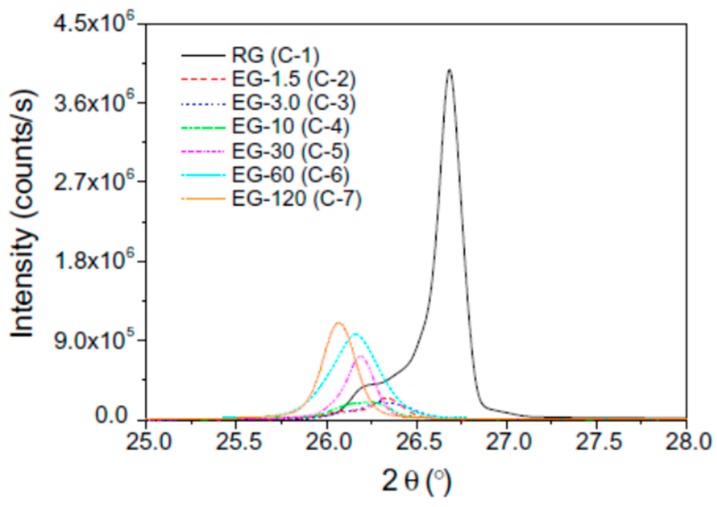
X-ray diffraction patterns of the RG (C-1) and EGs as a function of the heat treatment time (C-2 to C-7).

**Figure 6:**
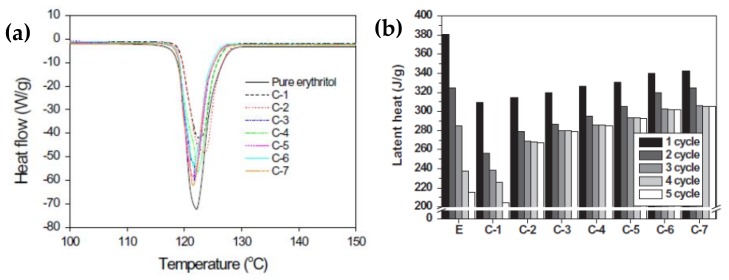
(**a**) DSC analysis of pure erythritol, the erythritol/RG composite, and erythritol/EG composites. (**b**) Changes in the latent heat of pure erythritol, the erythritol/RG composite, and erythritol/EG composites with thermal cycling.

Figure 5 shows the XRD patterns of the RG and EGs with different heat treatment time. The latent heat and thermal cycling stability were evaluated using a DSC tracer (Figure 6). As the Figure 6 shows, the thermal conductivity and latent heat increased as the distance between the EG layers increased. The thermal conductivity of the erythritol/EG composite with the largest EG layer spacing was 3.56 W/mK, and the latent heat value of pure erythritol was 90% by mass. The thermal cycling of the erythritol/EG composite was tested for five cycles and stabilized after the 3rd cycle, showing good thermal cycling stability. Table 4 lists the thermal properties of several prepared graphite-filled organic PCMs and compares them [89,94,95,98,99,100,101,102,103,104].

#### 3.2.3. Thermal Properties of Organic PCMs Filled with Other Carbon Materials

Carbon nanofibers (CNFs) can be used to enhance the performance of paraffin and soy wax-based energy storage materials [87]. Composite PCMs were synthesized through a mixed melting method using CNFs with different mass fractions as raw materials. The results show that CNFs can effectively improve the thermal conductivity of PCMs, and the thermal conductivity of composite PCM increases with the increase in the CNF mass ratio. The thermal properties of a paraffin-based nanocomposite PCM including CNFs were also investigated in another study, in which the CNF-PCM exhibited a thermal conductivity enhancement of up to 25.47% at 95 wt% loading [89]. Li et al [104] used amber and a variety of melons including winter melon and watermelon, and pyrolyzed them at 800 °C in a nitrogen atmosphere to prepare a carbon aerogel (CA). A black CA/paraffin composite was prepared by impregnating 95% by weight of paraffin wax into a CA substrate. The composites were reported to be desirable, thermally stable, lightweight, and environmentally friendly thermal storage materials with a stable melting–freezing enthalpy (115.2 KJ/Kg). Zhang et al. [105] reported the impregnation of octadecanoic acid (OA) into a graphene aerogel (GA) support material that was synthesized by the hydrothermal treatment of graphene oxide. Because of the lower bulk density of GA, the weight percent of GA in the composite was lower (approximately 15%). Thus, the composite could provide a high heat storage capacity of 181.8 J/g, which is very close to the OA’s individual heat storage capacity (186.1 J/g). The GA/OA composite had a higher thermal conductivity (2.635 W/mK) at a GA loading fraction of 20 vol%, which was approximately 14 times that of OA (0.184 W/mK). Table 5 lists and compares the thermal properties of several organic PCMs filled with different types of carbon materials [87,89,96,104,105,106,107,108,109].

## 4. Applications of PCMs

With the development and advancement of technology, PCMs have been applied in various fields, such as construction, solar energy, space industry, textiles, and others [110].

The PCMs are used in construction to meet the thermal comfort standard, which means that the phase transition temperature of the adsorbed paraffin should be 18 °C and 36 °C. In addition, chemical stability and other properties, fire characteristics, and compatibility with building materials also need to be considered. Phase-change materials latent heat storage technology has been widely used in walls, ceilings, and floors. Solar energy is used for passive solar heating in sunshine hours, which plays an important role in reducing temperature fluctuations. It is also suitable for off-peak heat storage, ventilation, and cooling.

With an increasing number of issues related to fossil fuels and the environment, thermal energy storage for building heating and cooling has become increasingly important [111,112,113,114,115]. Heat storage is considered to be a major problem in the development of solar energy under various climatic conditions. Unlike traditional sensible heat storage methods, PCMs have a higher energy storage density and store and release heat at almost constant temperatures. Phase-change materials are available for both active and passive space heating and cooling systems. Phase-change materials for heating and cooling of water have been developed by many researchers [116,117,118,119]. Barba et al. [116] analyzed the performance of a sealed salt hydrate as a potential storage tank in a domestic hot water storage tank heat transfer system. The salt is a eutectic mixture of ammonium and magnesium hydrate nitrates with a lower melting temperature. In addition, the discharge process of the PCMs is analyzed and the phase transition is performed with a constant surface temperature. The discharge effects of the materials in three different geometries packaged in flat, cylindrical or spherical polyethylene containers were evaluated. The motion boundary model of phase change material during discharge and its duration are studied. In PCMs of different geometric configurations, it was found that for small spherical capsules with high Jacob number and high thermal conductivity, the complete solidification time was the shortest. Cabeza et al. [117] added a PCM module on top of the stratified storage tank. Lleida University established an experimental solar test plant to test the behavior of PCM under real conditions. It can work continuously with solar systems or with electric heaters. The geometry of the PCM module used is the use of several cylinders at the top of the tank. Experiments were performed on 2, 4, and 6 PCM modules in the actual installation. In this experiment, a granular PCM graphite composite material of about 90 volts % sodium acetate trihydrate and 10 volts % graphite was used as the PCM material. The experimental results show that integrating the PCM module into the domestic hot water tank is a very promising technology. It will allow long-term use of hot water without an external source of energy, or use a smaller tank to achieve the same goal.

Recently, latent functional thermal fluids (LFTFs) have attracted people’s attention due to its higher specific heat than that of conventional single-phase heat transfer fluid in the phase-change temperature range. Latent functional thermal fluids can be applied in various applications such as heating, cooling, ventilation, and heat exchange [120,121,122]. Considerable research has used PCMs in thermosolar energy systems in which heat is stored during the day for use at night [123,124,125]. For example, Gil et al. [124] used different PCM to prepare and test solar cooling systems. A high temperature test plant capable of testing different types of thermal energy storage systems and materials was designed and built at the University of Leeds, Spain. The test plant is mainly composed of a heating system, a cooling system, and different storage tanks. The pilot plant uses synthetic heat transfer oil as a heat transfer fluid (expenditure), operating temperatures ranging from 100 to 400 °C.

Phase-change materials are widely used in various smart textiles [126,127,128,129,130]. In the early 1980s, NASA synthesized a smart textile that included PCM microcapsules in spacesuits to improve the thermal insulation properties [126,129]. Electrical energy consumed in garments assembled with PCM under the same conditions was reported to be approximately 31% less than that in PCM-free garment assemblies [128]. Energy storage is a major aspect of space applications. Therefore, conducting various studies to investigate and predict the thermal performance of PCMs in space applications is of great significance [131,132,133,134].

## 5. Conclusions

Using the latent heat capacity of PCMs as a heat storage system is an effective means of storing heat. Over the years, extensive research has been conducted on this topic and several strategies have been considered to overcome the problems of using PCMs and to broaden the application prospects of this technology. This study reviewed the research progress of using carbon materials as organic PCM fillers to overcome the shortcomings of many organic PCMs materials such as high melting temperature or low thermal conductivity. It also reviewed and summarized current state-of-the-art developments regarding the research and application of organic PCMs filled with various carbon materials for TES applications.

## Figures and Tables

**Table 1:** Thermal physical properties of some paraffin materials.

Materials	Melting Point (°C)	Latent Heat (kJ/kg)
Tetradecane	5.9	258
Pentadecane	9.9	193.9
Hexadecane	18.1	236
Heptadecane	20.8	171
Icosane	36.7	246
Tricosane	47.5	232
Hexacosane	56.3	256
Nonacosane	63.4	240
Dotriacontane	69.5	170
Tetratriacontane	75.9	269

**Table 2:** Thermal physical properties of some fatty acids.

Materials	Melting Point (°C)	Latent Heat (kJ/kg)
Capric acid	30.1	158
Lauric acid	43.7	210.8
Pentadecanoic acid	52.5	158.6
Myristic acid	52.1	190
Palmitic acid	54.1	183
Stearic acid	64.5	196

**Table 3:** Thermal properties of organic PCMs filled with carbon nanotubes.

PCMs	Melting point Temperature (°C)	Thermal Conductivity (W/mK)	Thermal Conductivity Enhancement (%)
Soy wax/CNTs [87]	37	0.403	24.38
n-Octadecylamine/f-MWCNTs [88]	54.6	0.532	86.66
Paraffin/S-MWCNTs [89]	57.9	0.43	63.49
Paraffin/L-MWCNTs [89]	58.4	0.39	48.28
Paraffin/Cu_2_O–Cu-MWCNTs [90]	52.32	0.34	17.24
Palmitic acid-stearic acid/carbon nanotubes (CNTs) [91]	53.59	0.341	29.7
Stearic acid/acid treated carbon nanotubes (a-CNTs) [92]	22	NA	NA
PEG6000/SiO_2_/CNTs [93]	53.3	0.421	51.3
PEG10000-co-N,N′-dihydroxyethyl aniline/surface-modified SWCNTs [94]	31.5	0.334	25.09

**Table 4:** Thermal properties of organic PCMs filled with graphite derivatives.

PCMs	Melting Point (°C)	Thermal Conductivity (W/mK)	Thermal Conductivity Enhancement (%)
Paraffin/GNP [89]	57.9	0.7	166.15
Paraffin/graphene [94]	NA	0.5	100
Paraffin/xGNP [95]	54.9	2.4	805.66
Paraffin-oleic acid (eutectic gel)/nanographite [98]	53.5	0.662	264
Palmitic acid/polyaniline (PANI)/xGNP [99]	61.57	1.08	237.5
Erythritol/EG [100]	116.32	2.06	564.5
Erythritol/EG [101]	NA	3.56	401.4
Palmitic acid/graphene nanoplatelets [102]	61.16	2.11	776.19
Stearic acid/graphene oxide [103]	32.57	NA	NA
Palmitic acid/nitrogen doped graphene [104]	66.52	1.73	517.85

**Table 5:** Thermal properties of organic PCMs filled with other carbon materials.

PCMs	Melting Point Temperature (°C)	Thermal Conductivity (W/mK)	Thermal Conductivity Enhancement (%)
Paraffin wax/CNFs [87]	50	0.45	40.62
Paraffin/CNFs [89]	58.4	0.33	25.47
Paraffin/carbon aerogel [104]	53.5	NA	NA
Octadecanoic acid/graphene aerogel [105]	57	2.635	1332.06
Paraffin/3D graphene foam [106]	58	0.617	99.67
Lauric acid/activated carbon [107]	44.07	0.18	12.5
Palmitic acid/carbonized alkylated silica aerogel [108]	61	NA	NA
Octadecanol/carbonized alkylated silica aerogel [108]	59	NA	NA
PEG1500/active carbon [109]	48	NA	NA
PEG10000-co-N,N′-dihydrox-yethyl aniline/carbon black [96]	29.3	0.280	4.86
